# Availability and consumer experience of mental health crisis services in Australia

**DOI:** 10.1177/10398562251379957

**Published:** 2025-09-14

**Authors:** Ryan Blasic, Vinay Lakra

**Affiliations:** 2281 Melbourne Medical School, University of Melbourne, Melbourne, VIC, Australia; Department of Psychiatry, University of Melbourne, Melbourne, VIC, Australia; Mental Health Division, Northern Health, Melbourne, VIC, Australia

**Keywords:** mental health, crisis, crisis intervention, Australia

## Abstract

**Objective:**

The number of individuals presenting with a mental health crisis in Australia has been steadily increasing. This review aims to identify what crisis intervention services are available in Australia for consumers experiencing a mental health crisis without an underlying severe psychiatric illness, and to explore the experiences of consumers utilizing these services.

**Method:**

Using PRISMA methodology, we conducted a review of four databases (Ovid Medline, PsycINFO, Web of Science, and Scopus) for articles relating to lived experience of consumers in Australian crisis intervention services. Inclusion and exclusion criteria were applied, and relevant articles assessed.

**Results:**

Eleven articles met the inclusion criteria. Identified crisis services included emergency departments, emergency services, crisis helplines, and community centres. Persistent themes included negative attitudes from staff, discordance around what constitutes a mental health crisis warranting presentation, long wait times, and the role of these services in providing non-clinical emotional support rather than medical interventions.

**Conclusion:**

This review identified clear discrepancies in consumer experiences between services, with specific aspects of care consistently linked to either positive or negative experiences. Improving staff communication and addressing judgemental attitudes may improve experiences. System reform would be better guided by further research into why consumers engage with specific crisis services.

Mental health related emergency department (ED) presentations have been steadily increasing in Australia over the past decade.^1^ In 2022-23, there were almost 300,000 such presentations, accounting for approximately 3% of all Australian ED visits.^2^ The most common reasons for a mental health presentation related to stress and substance use.^2^ This increased service demand has resulted in extended wait times for consumers as hospital services struggle to manage the influx.^1^

Despite the increasing demand, there is growing international evidence suggesting that EDs are not always the most appropriate setting for mental health presentations. Numerous studies have suggested that EDs are often poorly equipped to support individuals experiencing a mental health crisis, for a variety of reasons.^3–6^ While the concept of a mental health crisis is often complex and difficult to define, this review will define it as the presence of emotional distress beyond the coping mechanisms of the individual.^7^ For consumers experiencing a mental health crisis characterised by a relapse of a severe psychiatric illness, EDs may be appropriate settings, as these individuals often require medical intervention and potentially psychiatric inpatient admission. However, a significant proportion of people experiencing a crisis do not have any underlying severe psychiatric illness.^1,8^ These crises often result from psychosocial stressors and suicidal ideation. For this population, the ED is less suitable, as they can often be effectively managed in a less clinical environment.^1,9^

The recent Royal Commission into Victoria’s mental health system highlighted the importance of providing a range of alternative services to the ED that are better suited to supporting consumers during a mental health crisis.^1^ Currently, there are a variety of governmental and non-governmental services available for someone in crisis to access in Australia.^10^ These include the ED, police and ambulances services, crisis assessment and treatment teams (CATT), helplines such as Lifeline or Beyond Blue, and a select number of community services and organisations.

Existing literature on mental health crisis pathways predominately focuses on individual service models in isolation, with limited comparative analysis across multiple pathways. As such, it is currently unclear whether inequalities exist between these services, or if consumers would benefit from engaging with one service over another. Additionally, the literature is largely quantitative, emphasising service efficacy through metrics such as reductions in ED presentations and clinical outcomes. There is limited qualitative research about consumers lived experiences utilising these services. The Royal Commission emphasised that insufficient consideration of the consumer experience acts as a barrier preventing service improvement and reform.^1^ Therefore, the aim of this review is to identify what crisis interventions services are available in Australia for consumers without an underlying severe psychiatric illness during a mental health crisis and to explore the lived experiences of the consumers utilising these services.

## Method

This review followed PRISMA guidelines and involved the systematic search of the four online databases, Ovid Medline, PsycINFO, Web of Science, and Scopus, for full text articles relating to the lived experience of individuals utilising mental health crisis intervention services. The databases were accessed between January–March 2025. Key words utilised in the search included mental health crisis, mental health service, psychiatric service, crisis intervention, crisis support, lived experience, and Australia.

**Figure 1.**
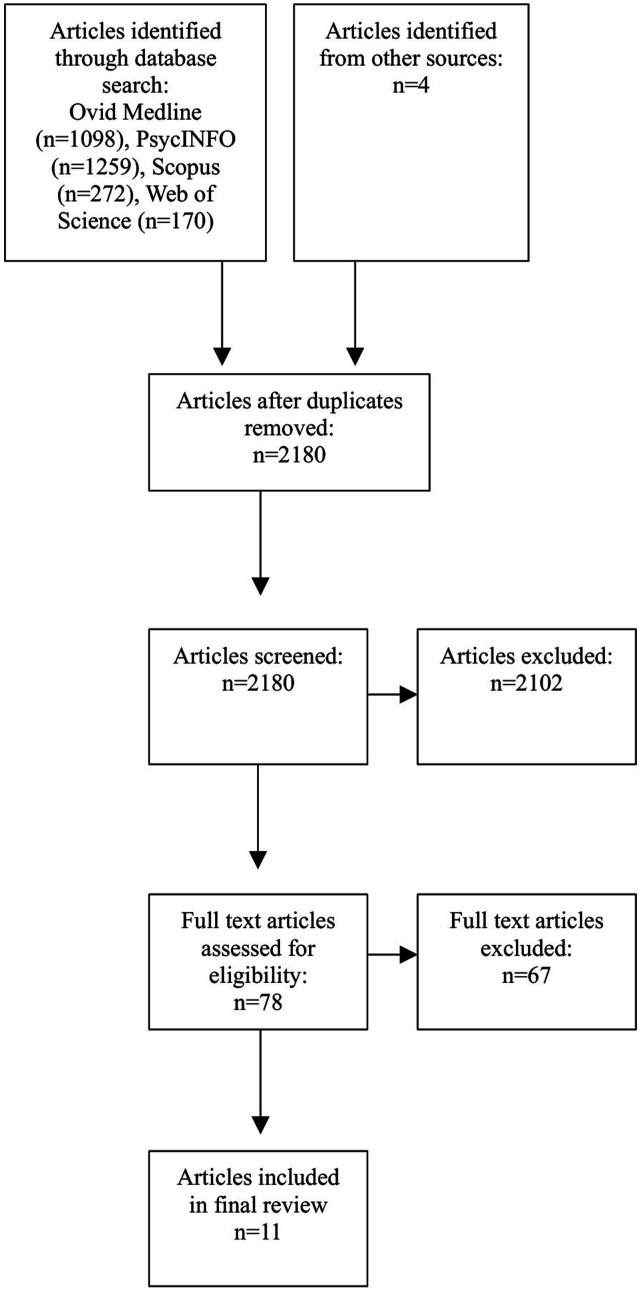
PRISMA flow diagram.

To be included in this review, the articles had to contain the lived experience of consumers in Australia and pertain specifically to crisis intervention services. Articles were limited to those written in English. Articles about consumers’ lived experience within the mental health system in general were excluded, as were articles which represented the lived experience through quantitative data such as satisfaction scores. Articles which focussed on consumers with significant or severe psychiatric illness were also excluded (Figure 1).

## Results

Eleven articles met the criteria for inclusion in this review. Two discussed experiences of consumers in ED specifically, four related to contact with emergency services (police, ambulance, CATT), three discussed helplines, and one referred to community crisis services. One article discussed multiple service experiences including ED, helplines, and community services (Table 1).

**Table 1.** Summary of included articles.

Reference	Crisis intervention service	Participants	Location	Information gathering approach	Results/Conclusions
Andrew et al., 2023	Emergency department	7 consumers (5F, 2M)	Western Australia	Focus groups	All participants reported negative experiences in ED, describing it as too ‘clinical’ and impersonal, which was perceived as disempowering. They also reported feeling judged and stigmatised due to the mental health nature of their presentation. Consumers described attending ED as a last resort due to a lack of other options, especially at nighttime. Furthermore, they describe an ideal safe space as non-clinical and inclusive, with peer support workers and staff trained in mental health crisis care.
Boscarato et al., 2014	Emergency services	11 consumers (5F, 6M)	Melbourne, Victoria	Semi-structured interviews	Consumers had mixed experiences with emergency services. All consumers spoke negatively of police-only responses, stating police were threatening and often exacerbated distress. Experiences with CATT were more positive. Consumers stated that positive interactions had been timely, respectful, non-judgemental, and staff had actively listened. Overall, emergency services were not the preferred crisis intervention service of any consumer, with a preference for informal support from family/friends, or if necessary, GPs
Bradbury et al., 2017	Emergency services	16 participants (6 consumers, 4 carers, 6 service providers)	New South Wales	Semi-structured interviews	Consumers who were taken into police custardy during a mental health crisis felt they were treated like criminals and humiliated. They described officers as disproportionately threatening and forceful. Consumers all agreed that positive interactions had been non-violent, compassionate, and employed good communication.
Evangelista et al., 2016	Emergency services	12 consumers	Melbourne, Victoria	Semi-structured interviews	Consumers praised CATT for their timely, persistent, and considerate response. This was compared to a police-only response, which consumers described as forceful, inadequate and lacking empathy.
Innes et al., 2025	Emergency department	42 consumers (26F, 16M)	Melbourne, Victoria	Semi structured interview	Consumers reported experiencing long wait times without receiving care. Staff were often dismissive of their concerns, downplaying the severity of the mental health crisis. Consumers also expressed feeling judged and unsafe in ED. They felt their experience would have been improved by shorter wait times, more compassionate staff, and improved communication.
Middleton et al., 2016	Helplines	19 callers to Lifeline (12F, 7M)	-	Semi structured interviews	Consumers reported varied, but mostly positive experiences with Lifeline. They appreciated that it was a non-specific service, and thus able to assist with a variety of mental health concerns. Additionally, being easily accessible and available 24 hours a day was praised. Positive interactions involved consumers feeling that the staff listened to them. Negative interactions involved staff being rigid with rules and risk assessments, rather than having a conversation with the consumer.
Randall et al., 2025	Emergency services	20 consumers (12F, 4M, 4 other)	Victoria, New South Wales, Queensland, Tasmania, Australian Capital Territory	Semi structured interviews	Consumers report largely negative interactions with the police. They found police often increased distress and escalated situations. Use of force was common and described as excessive. Additionally, the interactions with police left consumers feeling shame and humiliation. Positive interactions with police involved officers showing compassion and listening to consumers. Consumers overwhelming preferred police to not be involved in mental health crises.
Roennfeldt et al., 2024	Emergency department helplines community services	31 consumers (20F, 7M, 4 other)	-	Interviews	ED: Experiences were overwhelming negative. The clinical environment exacerbated distress, while consumers felt staff were often dismissive of mental health presentations, leading to them feeling unsafe. Helplines: Consumers appreciated the availability of helplines and found having someone to talk to useful. However, they also found that, at times, helplines were too focussed on risk assessment, rather than listening to the consumer. Overall, experiences were mixed, but more positive. Community: Consumers appreciated that these services often employed peer support workers, who could understand their experiences. These services tended to be non-pathologizing of their presentation, and more compassionate. Overall, consumer experiences in these community crisis services were overwhelmingly positive. Consumers did note however that these services were often not suited for someone in acute crisis.
Sands et al., 2016	Helplines	64 consumers	Melbourne, Victoria	Interviews	Consumer experiences with the service were mixed. Positive experiences noted the availability of the service, as it was 24 hours. They noted that staff were considerate, caring, listened well, and offered meaningful assistance. Negative feedback related to the time taken to receive care, differing opinions on what constituted a crisis, and judgemental and dismissive attitudes from staff.
Trail et al., 2022	Helplines	100 male consumers	-	Interviews	Experiences with helplines were largely positive, with a few exceptions. Positive experiences were characterised by helpful counsellors, who listened to the consumer without judgement and deescalated emotional distress. In contrast, negative experiences involved consumers feeling they were not being taken seriously, with dismissive staff who were more focussed on risk management than the consumers’ experience. Additionally, consumers appreciated the 24-h nature of the service but were disappointed with wait times.
Safer care Victoria	Community service	Consumers who attended the Safe Haven Café	Melbourne, Victoria	Informal discussion	Consumers appreciated a non-clinical alternative to ED that did not focus on diagnosis or assessment. They felt safe and welcome, without fear of judgement. Consumers valued peer support staff with lived experience who understood their situation. Additionally, consumers were able to progress at their own pace, without being rushed along like in ED.

### Emergency department

Three articles^7,11,12^ discussed the experience of consumers who accessed ED during a mental health crisis in Australia. The responses of participants were overwhelmingly negative, with the majority of consumers describing ED as inadequate and poorly equipped for dealing with psychological distress, often worsening a crisis rather than resolving it.

The negative themes described by consumers were consistent across all three studies. Many described attending ED as a last resort, especially at nighttime. Consumers felt unsure of where else to access support during a crisis, especially when alternative support systems or services were unavailable. In the ED, consumers reported long wait times before receiving treatment, which contributed to feelings of being ignored. This exacerbated distress and resulted in consumers feeling unsafe. Additionally, the clinical and impersonal environment of ED further exacerbated feelings of isolation and fear. Staff were frequently described as judgemental, dismissive, and downplaying of the severity of crises. This left consumers feeling stigmatised and disempowered, believing that staff were not taking them seriously.

In addition to the experiences in ED, Andrews et al.^11^ and Innes et al.^12^ explored consumers’ opinions on how to improve the ED to better cater to those in crisis. Common suggestions included improved training for staff regarding mental health presentations, clear and effective communication, and non-judgemental attitudes. Consumers in both studies also strongly advocated for the inclusion of peer support staff in ED, believing these individuals with lived experience would be better able to advocate for them and their needs.

### Emergency services

Four articles^13–16^ detailed consumer interaction with emergency services, including police, paramedics, and CATT. Police interactions were discussed in all four articles, with consumers describing their experiences as overwhelmingly negative. The police response was seen as distressing and often escalated the situation. Officers were characterised as threatening, coercive, and at times violent. Many consumers described being forcibly restrained by officers, which left them feeling humiliated and like criminals. Additionally, consumers felt the police response often lacked empathy and compassion, with officers displaying judgemental attitudes towards the consumers. Randall et al.^16^ and Boscarato et al.^13^ specifically reported that consumers overwhelmingly did not want police involved in a mental health crisis response. Positive experiences with the police, while very limited, involved officers showing compassion and listening to the consumer without resorting to violence and physical restraint.

Boscarato et al.^13^ and Evangelista et al.^15^ detailed consumer experiences with CATT. They reported mixed, but mostly positive, interactions. Consumers praised CATTs ability to deescalate the situation through a timely, compassionate, and respectful response. When compared to interactions with police, consumers described CATT as more likely to listen to their concerns in a respectful way. Members of the CATT were viewed as less judgemental and displayed more empathy for the consumer and their situation. Negative experiences with CATT were similar to those with police, in that consumers felt staff were dismissive, rude, and judgemental.

### Helplines

Four articles^7,17–19^ explored consumer experience with Australian crisis helplines. Middleton et al.^17^ looked specifically at consumer experience with Lifeline, while the other articles did not specify a service. Overall, they reported mixed, but largely positive, experiences. Common themes that emerged included consumers’ appreciation for the 24-h availability of helplines, as it allowed crisis support to be accessed at any time. Additionally, the convenience of being able to access help without having to leave the house was also appreciated. While the accessibility of the service was praised, a number of consumers noted extended wait times before being connected to support staff.

Consumer satisfaction with helpline services was largely influenced by how well they connected with the staff member they spoke to. Users who reported positive interactions described staff as caring and considerate. They listened to the consumer’s perspective without judgement, were able to deescalate emotional distress, and offer meaningful assistance. In contrast, negative interactions involved staff being described as judgemental or dismissive of the consumer’s crisis. A contributing factor to these feelings was staff’s perceived inflexibility with risk assessment and management. Many consumers felt that staff were overly focussed on completing a risk screening and prioritised it over addressing the consumer’s concerns. This resulted in consumers feeling that they were not being taken seriously by staff.

### Community

Two articles^7,20^ discussed consumer experience with community crisis services. Safer Care Victoria^20^ focussed specifically on St Vincent’s Safe Haven Café, while Roennfeldt et al.^7^ did not specify a service. Consumers who attended community services appreciated having a non-clinical alternative to the ED and valued the non-pathologizing approach, which did not focus on diagnoses or assessments. Community services were more personal and provided meaningful human connection, which was lacking in the clinical environment of other crisis interventions. This cultivated a welcoming and inclusive space, which consumers could access without fear or judgement. The calmer environment also allowed consumers to navigate their crisis at their own pace, which was described as empowering.

The employment of peer support workers was also praised by consumers. The presence of individuals with lived experience gave consumers the opportunity to discuss their crisis with someone who could relate to their situation. Consumers in Roennfeldt et al.^7^ however did note that these community services were not always best suited for assisting people experiencing a significant mental health crisis.

## Discussion

This review of mental health crisis support services available in Australia demonstrates not just the variety of services available, but also the spectrum of experiences that consumers have had with these services. The studies included consumers from most states and territories in Australia, including both rural and metropolitan locations, thus providing insight from a geographically diverse Australian population. However, the studies were mostly limited to English speaking participants, and therefore results may not be representative of the experiences of non-English speaking consumers. Out of the four crisis support modalities discussed, emergency services, especially police, received the worst feedback from consumers. Officers were frequently characterised as threatening and intimidating, leaving consumers feeling humiliated and distressed.^13–16^ Consumers who attended an ED also reported largely negative experiences. The clinical and impersonal environment of the hospital left consumers feeling isolated and disempowered, while staff were often judgemental and dismissive.^7,11,12^ Australian crisis helpline receives similar criticism as the ED; however, overall consumers reported more positive and helpful experiences, and staff were described as more compassionate.^7,17–19^ Community crisis services, in contrast to the other three, received overwhelmingly positive feedback from consumers. These services were described as respectful, considerate, and understanding of the consumers’ situation.^7,20^

Analysis of the consumer experiences revealed four recurrent themes across all services. These include negative attitudes from staff, a discordance around what constitutes a mental health crisis warranting presentation to these services, long wait times when accessing help, and the role of these services as providers of non-clinical emotional support rather than medical interventions.

The majority of the negative experiences described by consumers related to how staff treated them. Interactions were frequently described as being judgemental and lacking empathy. Many consumers felt their crisis was not being taken seriously by the staff who they had turned to for help, resulting in many consumers reporting that they felt unsafe. This negative staff behaviour was reported most consistently by consumers who interacted with the ED and police; however, it was also reported to a lesser extent by those who accessed crisis helplines and community services. The experiences of consumers in this review are consistent with current literature^1,4,21^ relating to staff behaviour in mental health services in Australia. The recent Royal Commission in Victoria^1^ found that judgemental and dismissive attitudes from staff were not just occurring in crisis care services but throughout all levels of mental health care. This suggests that there is still a significant stigma surrounding mental illness in Australia, which in turn creates barriers for consumers accessing care. It has been shown that negative experiences can lead to disengagement from the system, resulting in consumers delaying presentation for mental health support until they are experiencing an acute crisis.^22^ This in turn places a greater burden on these mental health crisis services and likely results in poorer outcomes for consumers.

The second common theme that emerged from the articles is a discordance between what consumers and staff believe constituted a mental health crisis. Consumers felt staff minimised their experiences and attempted to downplay the severity of their crisis, leaving them feeling dismissed and ignored. Users of helplines and ED often reported that their perception of their crisis was not shared by service staff. Current literature emphasises the multifaceted and highly subjective nature of mental health crises and describes the broad spectrum of what an individual may experience during a crisis.^23,24^ This subjectivity can make it difficult to clearly articulate their experiences to care staff.^24^ When combined with a lack of visible physical symptoms, consumers are often left feeling like they struggled to convince providers of the severity of their condition.^25^ Staff not perceiving the consumer as being in a mental health crisis may have contributed to the judgemental and dismissive attitudes described by consumers above. This suggests that there is a disconnect between what mental health consumers in Australia view as a crisis, and what care providers deem an appropriate time to present to crisis support services. Further education of crisis support staff may therefore be needed to enhance understanding of why consumers may seek support, and the difficulties they may face when explaining their situation.

A third recurrent theme was the extended wait times experienced by consumers when accessing crisis support services. Users of helplines and ED frequently reported long delays before receiving care, which negatively impacted their overall experience. Research has shown that, on average, mental health presentations in ED wait significantly longer than non-mental health presentations.^1,2^ One contributing factor may be the dismissive attitudes of staff, and the differing interpretations of a mental health crisis discussed above. However, current literature^1,2,26^ has also shown that both ED and crisis lines have seen increased demand in recent years, and these extended wait times likely indicate that these services are struggling to meet the increased consumer demand. This suggests a growing need to expand these services to reduce wait times and improve the experience of those utilising the service.

The final theme identified is the role of these services as safe outlets for consumers to voice their distress, without requiring medical intervention. Across all four service pathways, consumers consistently described positive experiences when staff simply provided space to talk. Consumers felt most supported when staff took the time to listen to the consumer’s concerns without interruption, validated their perspectives, and made them feel seen and heard. This positive response was frequently achieved without providing any additional services to the consumer, beyond the opportunity to speak openly in a safe environment. This suggests that, for consumers in crisis without significant psychiatric illness, the primary value of these services lies in offering a supportive space to express emotions and discuss stressors, rather than delivering medical care or other tangible interventions. Community crisis services most frequently displayed this, with consumers who utilised these services reporting overwhelmingly positive experiences.^7,20^ One possible explanation for this is the presence of peer support workers with lived experience at these community locations. Consumers highlighted the importance of peer workers in providing a safe and understanding environment, and research^27,28^ has shown peer support workers to be effective in improving satisfaction of consumers utilising a service. This potentially demonstrates a need for peer workers to be present at all crisis support services, to improve the consumer experience, and encourage engagement with the services.^29^

### Strengths and limitations

This review’s strength is that it explores the experiences of consumers presenting in crisis to a wide variety of mental health services in Australia. This is a critically important topic, given the increasing demand on these services and the potential for this research to support reform to improve the consumer experience.^1,2^ Additionally, the inclusion of Australian specific studies in this review allows the findings to be directly applied to the Australian mental health system.

However, this review was limited by the number of available studies for each service, especially community crisis services. Although a wide range of services exists,^10^ including multiple helplines and community services, this review was only able to explore the experiences of a limited number, due to a lack of available data. As such, experiences reported may not be entirely representative of each crisis intervention modality. Similarly, the small sample size in the majority of the reported studies is another limitation. Mental health crises are complex and heterogenous, with the needs and expectations of consumers varied.^23,24^ Thus, the small sample sizes reported in these studies may not be representative of the broader experiences of individuals utilising the service.

### Recommendations and future directions

Overall, the findings of this review support several recommendations for improving consumer experiences with crisis services. The consistently negative experiences of consumers with police suggest that police should have an extremely limited, if any, role in responding to mental health crises. Similarly, the overwhelmingly poor experiences of consumers who accessed ED implies that, without any clear indication for medical interventions, ED also has a limited role during mental health crises for consumers without an underlying psychiatric illness. These findings also highlight a need for further mental health education and training of staff to combat the stigma and discrimination that continues to be experienced by those in a mental health crisis.

Helplines, in contrast, received largely positive feedback but were hindered by long wait times. Expansion of these services could provide consumers with easily accessible support, which consumers valued, while also alleviating the strain currently on the system and reducing wait times. One possible strategy that has shown promise internationally is the integration of AI chatbots and messaging services for crisis support, such as the *Mindline* project in Singapore, which has seen positive impacts in triage, counselling, and referral processes.^30^

While this review has highlighted the value of non-clinical crisis services in the community and the inclusion of peer support workers, there is still a paucity of data relating to consumer experiences in community services. The Victorian Government has made attempts to expand such supports for less severe crises with the recent rollout of several Mental Health and Wellbeing Hubs and Locals.^31^ However, these centres have not yet been evaluated for efficacy or for consumer satisfaction. As such, further research into the experience of consumers utilising community support services is necessary to produce a clearer guide on how best to further develop this service.

Finally, while several studies in this review included consumers from rural locations and First Nations consumers, the experiences of these groups were not specifically analysed. Both First Nations consumers and those in rural areas are significant users of mental health crisis services,^2,26^ yet there is little data on their specific experiences. Non-English-speaking consumers were also largely excluded from the studies included in this review, highlighting a similar gap. Therefore, further research into the experiences of these groups is needed to determine whether crisis intervention services are meeting the needs of Australia’s diverse consumer population.

## Conclusion

The aim of this review was to assess the current services available in Australia for consumers to access during a mental health crisis, and to explore their experiences with these services. Despite small sample sizes and paucity of data for a number of services, it is clear that there is a discrepancy in consumer experience between services. The services which provide a safe place with respectful and compassionate staff were preferred by consumers, while the services which were dismissive, judgemental, and minimised the consumer’s experiences were heavily criticised. The mental health sector in Australia would benefit from further research into the experiences of diverse consumers accessing mental health crisis support.

## Data Availability

No new data were created or analysed in this study. Data sharing is not applicable to this article*.
